# Audit of the Acute Management of Renal Colic in District Hospitals Within a National Health Service Trust

**DOI:** 10.7759/cureus.69825

**Published:** 2024-09-20

**Authors:** Habeeb Abdulrasheed, Ayokunle Adenipekun, Mohamed S Mohsin, Muhammad Ali Khattak, Waleed Elsayed, Haajra Cheema, Ivo Dukic

**Affiliations:** 1 Urology, University Hospitals Birmingham NHS Foundation Trust, Birmingham, GBR

**Keywords:** acute renal colic, medical expulsive therapy, non contrast ctkub, obstructive kidney stones, ureteric stent

## Abstract

Introduction: Renal colic is a prevalent acute urological emergency caused by urinary stones and commonly manifests as severe loin pain. This audit assesses the management of acute renal colic at a National Health Service (NHS) Trust in the West Midlands, England, comparing practices against the National Institute for Clinical Excellence (NICE) and the British Association of Urological Surgeons (BAUS) guidelines.

Method: This retrospective audit reviewed 417 patients with suspected renal colic over a month. Data collected included patients' demographics, time to complete CT scan, laboratory investigations requested and management modalities. Patients assessed were either admitted through the Emergency Department, Surgical Assessment Unit, or previously admitted for other conditions on the wards. The audit benchmarked against NICE and BAUS guidelines, focusing on diagnostic timeliness and management, including essential blood investigations. Data analysis performed using SPSS (IBM Corp., Armonk, NY, USA) included descriptive statistics and Chi-square tests, with significance set at p < 0.05.

Results: A total of 417 patients met the inclusion criteria. The average age of patients was 47.4 years with slightly higher male population (51.1%). The diagnostic rate for renal or ureteric stones using computed tomography of the kidneys, ureters, and bladder (CT-KUB) was 41.2%. The positive detection rate was significantly higher in males (54%) compared to females (27.5%) while females have more alternate diagnosis than males (13.7% vs 6.1%). The average completion time of CT-KUB was nine hours with 7.7% of patients having their CT-KUB beyond the 24-hour benchmark. Most patients (48%) were managed conservatively with analgesia, 32% received tamsulosin as medical expulsive therapy and others had interventions like ureteric stent insertion (9.3%), nephrostomy (6.4%) or primary ureteroscopy (4.1%).

Conclusion: The audit of renal colic management at our centre revealed a 41.2% diagnostic rate for renal or ureteric stones via CT-KUB, with a significantly higher rate in males than females. Diagnostic delays were minimal, with only 7.7% of scans exceeding 24 hours. Conservative management was common, however non-steroidal anti-inflammatory drugs (NSAIDs) were underutilized despite guideline recommendations. There was a notable gender disparity in diagnostic and alternate findings rates. The results showed the need for adherence to pain management protocols and highlight the effectiveness of existing renal colic protocol, while also pointing to potential areas for improvement in treatment uniformity and guideline adherence.

## Introduction

Renal or ureteric colic is an acute, severe, and sudden onset loin pain caused by the presence of an obstructing stone along the path between the kidneys and the urinary bladder [1]. It is one of the most common acute urological emergencies, with an annual incidence of one to two per 1,000 people [2]. About 12% of men and 6% of women will experience at least one episode at some point in their lives, with the peak incidence occurring between ages 40 to 60 for men and 20 to 30 for women [2].

Urinary stones are formed by the aggregation of urinary crystals with a non-crystalline protein matrix. These stones grow until they pass into the ureter, often causing obstruction and colic symptoms. Eighty percent of stones contain calcium, while uric acid and infection stones (magnesium ammonium phosphate) each account for approximately 7% [3].

The pain is caused by the spasm of the ureter around the stone and by the physical stretching of the ureter, pelvicalyceal system, and renal capsule due to the blockage. This leads to sudden severe pain, often accompanied by nausea, vomiting, and occasionally hypotension and fainting [3,4].

Immediate pain relief using non-steroidal anti-inflammatory drugs (NSAIDs) is the first treatment requirement in patients with suspected acute renal or ureteric colic, and this should not be delayed for imaging [2,5,6]. If NSAIDs are contraindicated or pain control is ineffective, intravenous paracetamol can be administered. Opioids do not offer superior benefits over NSAIDs and may lead to opioid dependence; hence, they are recommended only if pain control is not achieved with the NSAIDs or paracetamol [2].

A non-contrast-enhanced computed tomography of the kidneys, ureters, and bladder (CT-KUB) is the standard diagnostic tool for renal colic and should be performed within the first 14 to 24 hours [2,5]. Other imaging modalities, such as ultrasound scans of the urinary tract, are recommended for patients with contraindications to CT-KUB or to avoid radiation exposure, especially in pregnant women and children. In selected patients, ultrasound can have sensitivity close to that of CT-KUB [7,8].

Additional investigations required for the initial assessment of patients with renal colic include urine dipstick testing (with culture if indicated), serum creatinine and electrolytes (including estimated glomerular filtration rate (eGFR)), calcium, uric acid, full blood count (FBC), and C-reactive protein [2,5,6].

Medical expulsive therapy using alpha-blockers, such as tamsulosin, is recommended for patients with distal ureteric stones larger than 5 mm [6]. Primary stone treatment, either by ureteroscopy or shock wave lithotripsy, depending on stone characteristics and patient factors, should ideally be undertaken within 48 hours [2,5,6]. If immediate treatment is not possible, a stent may be placed, with subsequent ureteroscopy performed within four weeks to reduce morbidity [6]. Patients showing signs of an infected obstructed system require urgent decompression, either through retrograde stent insertion or percutaneous nephrostomy [6].

The study aims to compare the current management of patients with renal colic against the National Institute for Health and Care Excellence (NICE) and British Association of Urological Surgeons (BAUS) guidelines to assess our current practice and identify areas for improvement to enhance patient care and experience.

## Materials and methods

This is a retrospective audit of patients with suspected renal colic at University Hospital Birmingham NHS Foundation Trust over the course of a month. Data for the study was collected from October 1 to 31, 2023.

A total of 417 patients were included in the study. These patients were admitted either through the Emergency Department (ED) or the Surgical Assessment Unit (SAU), where CT-KUB was requested at the time of admission. A third group included patients who had CT-KUB requested for suspected renal colic while already admitted to the wards. All patients who underwent CT-KUB for renal colic in any of these departments within our hospitals within the period of the study were included in the audit. The exclusion criteria comprised patients who had CT-KUB done for reasons other than suspected renal colic.

The audit was registered with the hospital's Clinical Audit Registration System, and permission was granted to access patient data. Information collected included patient demographics, time to complete CT-KUB, and laboratory investigations requested (serum creatinine, full blood count, uric acid, calcium), which were sourced from the hospital's health informatics department.

Variables assessed in this audit include age, gender, time to CT-KUB (defined as the time from the request for CT-KUB to the reporting of the results), the ordering of essential laboratory investigations (serum urea and creatinine, full blood count, calcium, and uric acid) and urine dipstick, analgesia prescribed, and the treatment methods for patients with urinary tract stones.

The audit was benchmarked against the NICE and BAUS guidelines [2,5]. The NICE guidelines recommend that a CT-KUB should be performed within 24 hours of presentation. BAUS guidelines specify a 14-hour window but allow up to 24 hours in hospitals without 24-hour radiology facilities. Both NICE and BAUS advise that blood tests, including serum urea and creatinine, full blood count, calcium, and uric acid, should be requested at presentation. Additionally, they recommend NSAIDs as the first-line analgesia unless contraindicated. This audit was conducted in alignment with the BAUS national renal colic audit protocol [9].

Data was entered into Excel sheets (Microsoft, Redmond, WA, USA), and patient-identifying information was anonymized. After data cleaning, which involved removing duplicate entries, confirming adherence to inclusion criteria, and ensuring appropriate coding, descriptive and statistical analyses were conducted using SPSS version 29.0 (IBM Corp., Armonk, NY, USA).

Continuous variables, such as age and time to complete CT-KUB were reported as mean and standard deviation (SD). Categorical variables, such as gender were reported as counts and percentages. Statistical analysis was performed using the Chi-square test for categorical variables, with a p-value of 0.05 set for determining statistical significance.

## Results

Patient demographics

Four hundred and seventeen patients presented with suspected renal colic across sites and departments during the period. The average age was 47.4 years (SD=17.97). The youngest patient was 17 years, whilst the oldest was 91 years. There were 213 (51.1%) male and 204 female patients. Half of the CT-KUB scans (210, 50.4%) were requested at the ED, 24.7% (103) at SAU and 24.9% (104) on the wards. Details of patients’ characteristics are given in Table 1.

**Table 1:** Patient demographics The table represents patients' demographics expressed in numbers of patients (N) and percentages (%). P-value is significant at P<0.05. SAU, Surgical Assessment Unit; ED, Emergency Department; dof, degree of freedom

Patient demographics	Number of patients	Percentage (%)	Chi- square (X^2^)	P value
Age (years)				
17-30	88	21.1		
31-50	165	39.6		
51-79	100	24		
71-91	64	15.3		
Total	417	100	53.63 (dof= 3)	<0.001
Gender				
Male	213	51.1		
Female	204	48.9		
Total	417	100	0.194 (dof= 1)	0.66
Departments				
SAU	104	24.9		
ED	103	24.7		
Wards	210	50.4		
Total	417	100	54.42 (dof= 2)	<0.001

Investigations at presentation

**Figure 1:**
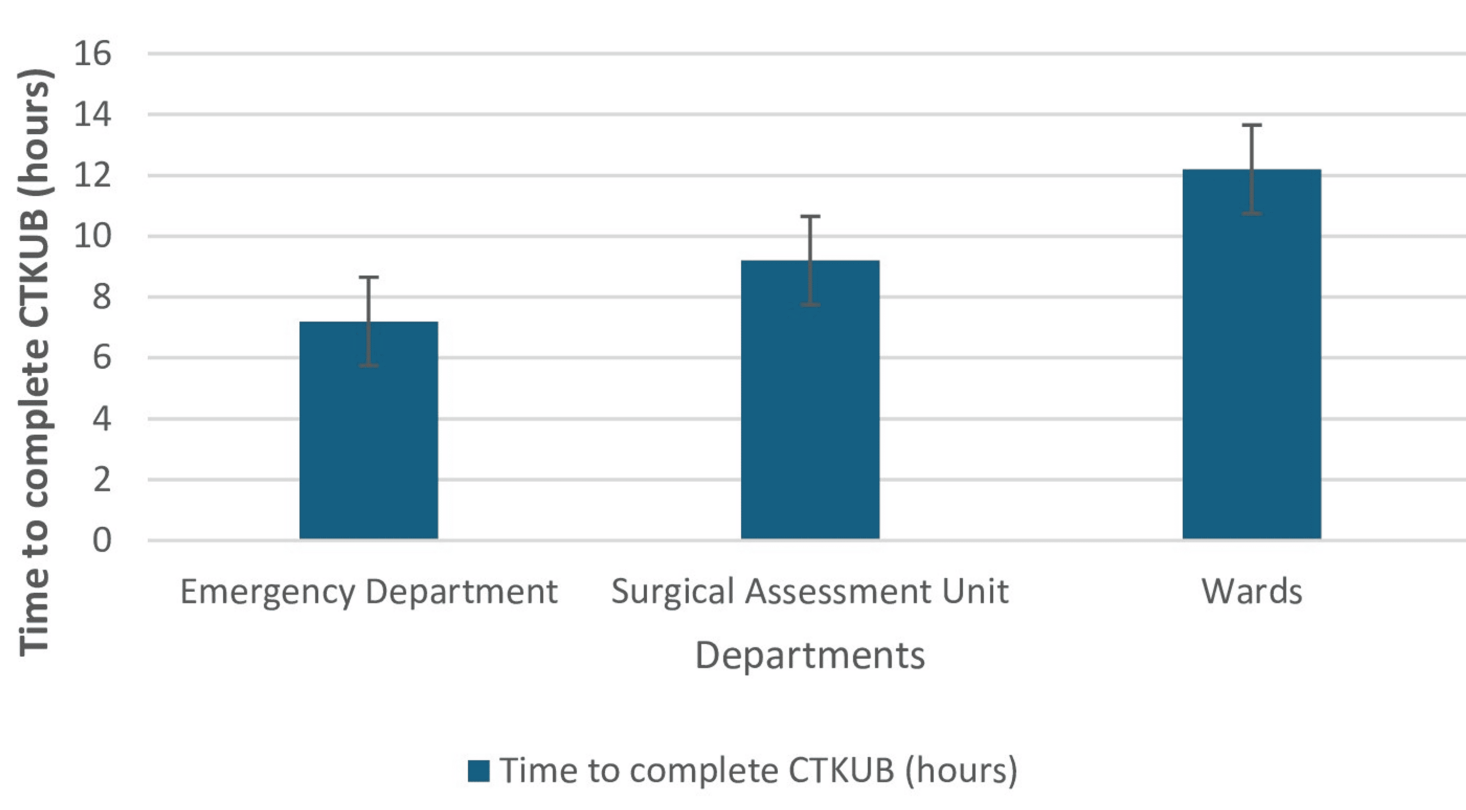
Time taken to complete CT-KUB across departments The data in the figure depicts the time taken to complete CT-KUB at different units represented in mean and standard deviation. CT-KUB, Computerised tomography scan of kidneys, ureters and bladder

The average time taken to time taken complete CT-KUB per patient was nine hours (SD 11.89). The longest duration was 29 hours and fastest was 30 minutes with 32 patients (7.7%) exceeding the 24-hour benchmark in guidelines. The average time taken to complete CT-KUB for a patient in ED, SAU and wards was seven, 11 and 12 hours respectively. A summary of the average time taken at different departments or units is depicted in Figure 1.

A total of 172 (41.2%) patients had stones in their urinary tract. Fifty-one patients had stones in the kidney, 20 in the proximal ureter, 19 in the mid-ureter, 26 in the distal ureter, and 40 at the vesicoureteric junction. The average stone size was 5.4 mm (SD, 3.42). Ninety-four of these patients (54.7%) had associated hydronephrosis and/or hydroureter, and 69 (40%) had associated fat stranding. The summary of the CT-KUB report and other investigations is presented in Table 2.

**Table 2:** Investigations done during patient assessment Investigations done and findings are represented in numbers (n) and percentages (%) in this table and Chi square calculated. P- value is significant at P<0.05. CT-KUB, Computerised tomography scan of kidneys, ureters and bladder; n, Number of patients; ED, Emergency Department; SAU, Surgical Assessment Unit; dof, degree of freedom

Stone detection rate on CT-KUB, n= 172		Chi- square (X^2^)	P value
By departments, n (%)			
ED	86 (50)		
SAU	44 (25.6)		
Wards	42 (24.4)		
		0.13 (dof= 2)	0.936
By gender, n (%)			
Male	115 (54)		
Female	56 (27.5)		
		38.48 (dof= 1)	0.001
CTKUB Findings			
Location of stone, n (%)			
Kidney	51 (29.7)		
Upper ureter	20 (11.6)		
Mid ureter	19 (11.0)		
Lower ureter	26 (15.1)		
Ureterovesical junction	40 (23.3)		
Bladder	16 (9.3)		
Total	172 (100)	33.58 (dof= 5)	<0.0001
Presence of Hydronephrosis/ Hydroureter n (%)			
Hydronephrosis only	14 (8.2)		
Hydroureter only	15 (8.7)		
Both	65 (37.8)		
None	78 (45.3)		
Total	172		
Laboratory investigations requested, n (%)			
Serum urea/creatinine	372 (89.2)		
Full blood count	370 (88.7)		
Serum uric acid	27 (6.5)		
Serum calcium	204 (48.9)		

The positive diagnostic rate of all CT-KUBs for renal or ureteric stones was 41.2% (172/417). A further 9.8% (41/417) had an alternate diagnosis. The positive rate in males was 54% (115/213) compared to that of females which was lower at 27.5% (56/204). This difference was found to be statistically significant (X² (1) = 35.48, p < 0.001).

**Figure 2:**
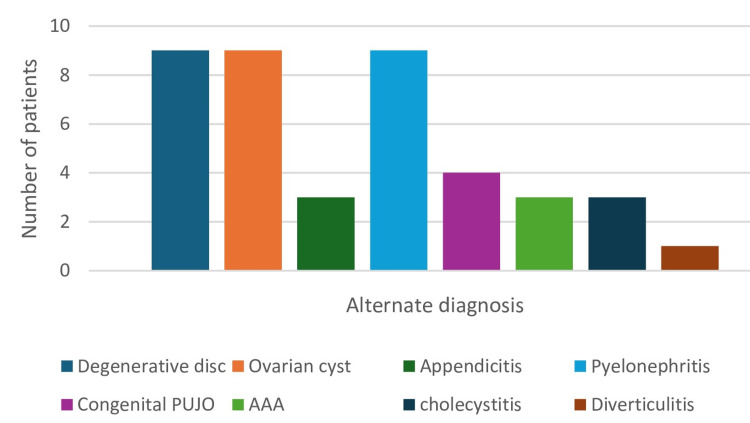
Alternate diagnoses on CT-KUB The figure shows significant alternate diagnosis to renal colic in the study represented in number of patients (n). CT-KUB, Computerised tomography scan of kidneys, ureters and bladder; AAA: Abdominal aortic aneurysm; PUJO: Pelvi-ureteric junction obstruction

The rate of other significant findings in males was 6.1% (13/213), lower than that of females which was 13.7% (28/204). The difference was also statistically significant (X² (1) = 6.79, p = 0.009). Common alternative diagnoses are depicted in Figure 2.

The ED had a diagnostic rate of 41% (86/210), similar to the SAU and the wards, which had diagnostic rates of 42.7% (44/103) and 40.4% (42/104), respectively. The differences in diagnostic rates across these departments were not statistically significant (X² (2) = 0.13, p = 0.936).

All patients had urine dip at presentation. About half of the patients (52.5%, 219/417) and 60% of those with CT-KUB-proven urolithiasis (103/172) had microscopic or visible haematuria at presentation.

Among the 417 patients who presented with suspected renal colic, 89.2% (372) had their serum creatinine and eGFR done. About 30% (127 patients) presented with reduction in renal function with serum creatinine rising by at least 26 micromol/liter above the upper limit (110 micromol/liter), but only about half (58 patients) was as a result of an obstructing stone.

Full blood count was done at presentation in 88.7% (370 patients). Of these patients, 106 (25.5%) had elevated white cell count (>11.00/mm3) and related to presence of an obstructing stone in only 38 patients.

Serum calcium and uric acid were done in 48.9% (204/417) and 6.5% (27/417) of patients respectively. Only a single patient had elevated calcium while uric acid was elevated in four patients.

Renal colic management

**Figure 3:**
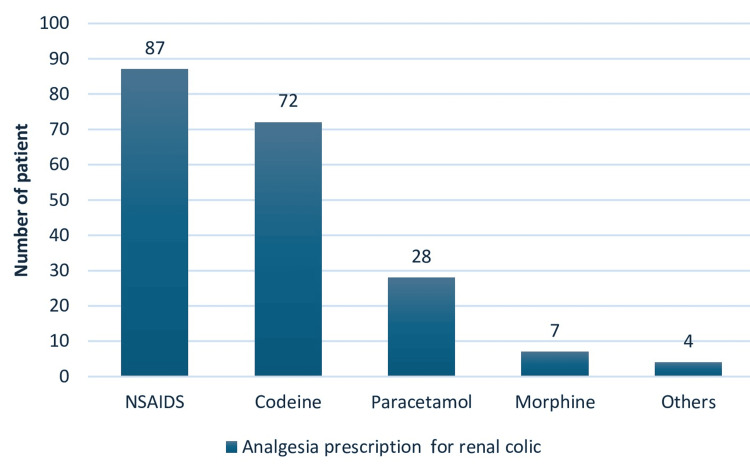
Analgesics prescribed for renal colic In this figure, analgesics prescribed to manage renal colic is represented in number of patients (n). NSAIDS, Nonsteroidal anti-inflammatory drugs; others, tramadol (2) hyoscine (2)

Analgesics given for renal colic were documented in 198 patients. Of all prescriptions reviewed, only 44% (87 prescriptions) were NSAIDs, 36% (73) were codeine, 14% (28) were paracetamol, 4% (7) were morphine and 1% (2) each for tramadol and hyoscine. Detail of medications prescribed for renal colic pain management is represented in Figure 3.

**Figure 4:**
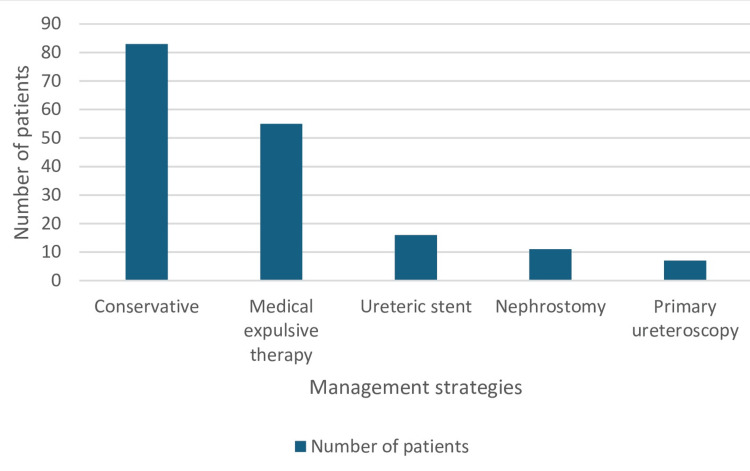
Management strategies for acute renal colic In this figure, the management strategies used in for patients with renal colic is represented in number (N) of patients.

About 48% of patients with CT-KUB-proven stones (83/172) had conservative management with analgesics and were discharged for follow-up at the stone clinic. Thirty-two percent (55/172) had medical expulsive therapy with tamsulosin 400 microgram, 9.3% (16/172) had ureteric stents, 6.4% (11/172) had nephrostomy and 4.1% (7/172) had primary ureteroscopy. Details of management are presented in Figure 4.

## Discussion

Patient demographics

Patient demographics in our audit were consistent with established epidemiological data on renal colic, showing a higher incidence in males and an average age between 40 and 50 years [10-12].

The diagnostic rate for renal or ureteric stones from all CT-KUB scans was 41.2% (172/417), with an additional 9.8% (41/417) having an alternative diagnosis. A similar retrospective study conducted in the United Kingdom reported an overall positive rate of 47.5% and 10.0% with significant alternative diagnoses [10]. However, a prospective study in France on patients with suspected uncomplicated renal colic found a higher diagnostic rate of 78%, with 10% having significant alternative diagnoses [11]. The variation in diagnostic rates may be due to differences in methodology and study populations.

In our audit, the positive rate for renal stones was higher in males (54%), which aligns with findings from another study reporting a male positive rate of 61.6% [10]. This is consistent with common knowledge that renal colic is more common in males than females [2,13].

Investigations requested

The average time to complete a CT scan was nine hours with only 7.7% exceeding the 24-hour benchmark. This may be a result of an existing local renal colic protocol across all hospital sites and departments.

CT-KUB detected stones in only 172 patients (41.2%). Most of these stones (61%) were found in the ureter and the ureterovesical junction is the most common location in the ureter seen in 38.1% of all ureteric stones (40/105). A similar study on the location of stones in ED patients with renal colic found that 60.6% of stones are found in the ureterovesical junction [14]. This is not surprising as the ureterovesical junction is one of the three areas of constrictions where ureteric stones could lodge and studies have proven that the ureterovesical junction is the commonest location of ureteric stones in patients presenting with ureteric colic [15].

All patients included in the audit had urine dipstick in compliance with both NICE and BAUS guidelines. About half of the patients with renal colic had microscopic or visible haematuria at presentation, and this proportion increased to 60% when only patients with CT-KUB-proven urolithiasis were considered. A similar pattern, though with higher percentages, was found in another study on the prevalence of microscopic haematuria, where 77% of all patients with renal colic and 84% of patients with urolithiasis had microscopic haematuria [16]. This confirms the common knowledge that not all patients with renal colic will have haematuria [17-19]. The reason for the difference in prevalence of haematuria may be due to difference in stone burden, severity of patients’ symptoms and sensitivity of diagnostic kits used.

NICE and BAUS guidelines recommended that all patients with renal colic should have full blood count, serum creatinine, calcium and uric acid at presentation [2,5]. In our study, investigations like serum creatinine and full blood count were done in 89.2% and 88.7%. However, serum calcium and uric acid were only done in 48.9 and 6.5%. This should be an area that requires improvement in our practice.

Management of renal colic

Most of our patients (48%) were managed conservatively and discharged home on analgesia only and followed up in the stone clinic. About 32% (55/172) had medical expulsive therapy with tamsulosin, an alpha-blocker. The NICE guideline recommends tamsulosin for distal ureteric stones less than 10mm [2]. BAUS discourages routine use of alpha-blockers like tamsulosin for expectant management because of controversial evidence in its support [5]. A multicentre placebo-controlled trial found that tamsulosin 400 μg and nifedipine 30 mg are not effective at decreasing the need for further treatment to achieve stone clearance in four weeks for patients with expectantly managed ureteric colic [20]. However, a more recent study on the role of alpha-blockers in ureteric stone management found alpha-blockers to be efficacious in the treatment of patients with much larger ureteric stones and who are amenable to conservative management [21].

About 9% and 6% of patients presenting with renal colic had ureteric stent insertion and nephrostomy insertion respectively to relieve obstruction in the acute setting. These patients were thereafter deferred for elective stone treatment.

The BAUS has recommended primary treatment within 48 hours of presentation or when not feasible immediately, ureteric stent may be inserted and stone treatment to be undertaken within four weeks of presentation [2]. However, only 4% of our patients had primary ureteroscopy in our centre. This may be a result of the unavailability of standard instruments for emergency laser tripsy in the hospital and most of these patients were deferred for elective stone treatment at another hospital site.

Limitations of this audit include a relatively short period (one month) and some missing data, such as analgesic prescriptions. We aim to address these limitations with a further re-audit over a longer period and with a larger sample and encouraging documentation of analgesics given.

## Conclusions

This audit demonstrates varying degrees of adherence to national guidelines in the management of patients with renal colic. While the time taken to complete CT-KUB in the hospital is within acceptable standards, much work needs to be done on investigation requests and analgesic prescription practice. Increasing the awareness of the local pathway and incorporating analgesics prescription into the current protocol will improve the practice and positively impact patient journey, therefore decreasing morbidity associated with renal colic.
